# Composition-Driven Ultra-Low Hysteresis Electrostrictive Strain in BaTiO_3_-BaZrO_3_-Bi(Zn_2/3_Nb_1/3_)O_3_ Ceramics with High Thermal Stability

**DOI:** 10.3390/ma19020374

**Published:** 2026-01-16

**Authors:** Xuyi Yang, Qinyi Chen, Qilong Xiao, Qiang Yang, Wenjuan Wu, Bo Wu, Hong Tao, Junjie Li, Xing Zhang, Yi Guo

**Affiliations:** 1 Information Materials and Device Applications Key Laboratory of Sichuan Provincial Universities, Chengdu University of Information Technology, Chengdu 610225, China; 2 Optoelectronic Sensor Devices and Systems Key Laboratory of Sichuan Provincial Universities, College of Optoelectronic Engineering (Chengdu IC Valley Industrial College), Chengdu University of Information Technology, Chengdu 610225, China; 3 Sichuan Province Key Laboratory of Information Materials, Southwest Minzu University, Chengdu 610041, China

**Keywords:** barium titanate, electrostrictive, low hysteresis, thermal stability

## Abstract

High electrostrain, excellent thermal stability, and low hysteresis are critical requirements for advanced high-precision actuators. However, simultaneously achieving these synergistic properties in lead-free ferroelectric ceramics remains a significant challenge. In this work, a targeted B-site doping strategy was employed to develop novel lead-free (0.99-*x*)BaTiO_3_-*x*BaZrO_3_-0.01Bi(Zn_2/3_Nb_1/3_)O_3_ (BT-*x*BZ-BZN, *x* = 0–0.2) ceramics. Systematic investigation identified optimal Zr^4+^ substitution at *x* = 0.1, which yielded an outstanding combination of electromechanical properties. For this optimal composition, a high unipolar electrostrain (*S*_max_ = 0.11%) was achieved at 50 kV/cm, accompanied by an ultra-low hysteresis (*H*_S_ = 1.9%). Concurrently, a large electrostrictive coefficient (*Q*_33_ = 0.0405 m^4^/C^2^) was determined, demonstrating excellent thermal robustness with less than 10% variation across a broad temperature range of 30–120 °C. This superior comprehensive performance is attributed to a composition-driven evolution from a long-range ferroelectric to a pseudocubic relaxor state. In this state, the dominant electrostrictive effect, propelled by reversible dynamics of polar nanoregions (PNRs), minimizes irreversible domain switching. These findings not only present BT-*x*BZ-BZN (*x* = 0.1) as a highly promising lead-free candidate for high-precision, low-loss actuator devices, but also provide a viable design strategy for developing high-performance electrostrictive materials with synergistic large strain and superior thermal stability.

## 1. Introduction

Piezoelectric actuators have attracted considerable attention for high-precision applications, such as optical positioning, micro-electromechanical systems (MEMSs), and fuel injectors, due to their instantaneous response, high generative force, and sub-nanometer resolution [1,2]. Ferroelectric ceramics, which convert electrical energy into mechanical displacement through electric-field-induced strain (electrostrain), act as the functional core of these devices [3]. In such systems, significant electrostrain is typically pursued through strategies like constructing morphotropic phase boundaries (MPB) or defect engineering. Currently, lead-based ceramics (e.g., Pb(Zr,Ti)O_3_) are widely utilized; however, their large strain primarily originates from extrinsic domain wall motion, which is intrinsically dissipative. This results in substantial strain-field hysteresis (*H*_S_) and control non-linearity [4]. Furthermore, the dual pressure of stringent environmental regulations and the demand for high-frequency precision have intensified the search for high-performance lead-free alternatives that can simultaneously deliver large strain, negligible hysteresis, and high thermal stability [5,6].

To overcome the hysteresis bottleneck, research has increasingly focused on the intrinsic electrostrictive effect, characterized by the longitudinal electrostrictive coefficient *Q*_33_ (*S*_33_ = *Q*_33_*P*^2^, where *S*_33_ denotes longitudinal strain and *P* represents polarization) [7,8,9,10,11]. Unlike ferroelectric domain switching, which involves significant energy loss, electrostriction stems from intrinsic lattice distortion and the reversible alignment of polar nanoregions (PNRs) within a relaxor matrix. This mechanism offers a pathway to achieve nearly hysteresis-free strain. Consequently, the core challenge lies in enhancing the *Q*_33_ coefficient while ensuring the material maintains performance stability across a broad temperature range [12].

BaTiO_3_ (BT) serves as a cornerstone lead-free ferroelectric due to its excellent dielectric properties and eco-friendliness, with recent research extending its utility from energy harvesting to advanced electronics [13,14,15,16,17]. Nevertheless, pure BT exhibits a stable tetragonal (T) phase at room temperature, characterized by strong long-range ferroelectric order. This results in high remnant polarization and large hysteresis, which is detrimental to precision control [18,19,20,21,22,23,24,25,26,27]. To bridge the gap between high strain and low loss, our strategy involves disrupting the long-range ferroelectric order of BT to drive it into a relaxor state. Recent studies indicate that BaTiO_3_-BiMeO_3_ solid solutions (where Me represents a trivalent or effectively trivalent cation) have proven effective in inducing relaxor behavior [28,29,30,31,32,33,34,35,36,37,38,39,40,41,42]. Among these, Bi(Zn_2/3_Nb_1/3_)O_3_ (BZN) is a key component; its heterovalent substitution on both A and B sites introduces intense local charge fluctuations and random fields, which are instrumental in fragmenting macroscopic domains into dynamic PNRs [39,40,41].

In this study, we propose a synergistic B-site doping strategy to further refine this relaxor behavior by constructing a ternary (0.99-*x*)BaTiO_3_-*x*BaZrO_3_-0.01Bi(Zn_2/3_Nb_1/3_)O_3_ (abbreviated as BT-*x*BZ-BZN) system. While BZN induces the initial relaxor state, the substitution of Ti^4+^ by the chemically more stable and larger Zr^4+^ acts to “pinch” the phase transition boundaries. This modification shifts the Curie temperature (*T*_C_) toward room temperature and stabilizes a pseudo-cubic (PC) symmetry, effectively intensifying chemical heterogeneity [42,43,44,45,46]. We hypothesize that this composition-driven evolution—coupling the random-field effect of BZN with the phase-pinching effect of BZ—will stabilize an ergodic relaxor state that suppresses irreversible domain wall motion while maintaining a superior *Q*_33_. The underlying mechanism of the large electrostrain is systematically investigated through unipolar/bipolar strain loops, X-ray diffraction, temperature-dependent dielectric behavior, and the thermal stability of the electrostrictive coefficient over the temperature range of 30–120 °C.

## 2. Materials and Methods

### 2.1. Material Preparation

(0.99-*x*)BaTiO_3_-*x*BaZrO_3_-0.01Bi(Zn_2/3_Nb_1/3_)O_3_ (abbreviated as BT-*x*BZ-BZN, with *x* = 0, 0.005, 0.008, 0.01, 0.015, 0.02, 0.1, 0.15, and 0.2) ceramics were fabricated via a conventional solid-state reaction method. High-purity raw powders, including Nb_2_O_5_ (99.5%), Bi_2_O_3_ (99%), BaCO_3_ (99%), TiO_2_ (98%), ZnO (99%), and ZrO_2_ (99%) (Sinopharm Chemical Reagent Co., Ltd., Shanghai, China), were weighed according to the stoichiometric formula and wet-milled in anhydrous ethanol for 10 h using a planetary ball mill. Subsequently, the dried powders were calcined at 1150 °C for 3 h. As a binder, 7 wt% polyvinyl alcohol (PVA) was added for granulation, and approximately 0.3 g of the powder was weighed and formed into disks (~10 mm in diameter) by dry pressing at 8 MPa. After burning out the PVA binder, the BT-*x*BZ-BZN samples were obtained by sintering at 1300 °C for 2 h. In preparation for dielectric and ferroelectric property measurements, the surfaces of the BT-*x*BZ-BZN samples were coated with silver paste and fired at 600 °C for 10 min.

### 2.2. Material Characterization

The crystal structures of the BT-*x*BZ-BZN samples were analyzed using an X-ray diffractometer (XRD) with Cu Kα radiation (Bruker D8 Advanced XRD, Bruker AXS Inc., Billerica, MA, USA). The surface morphologies were observed using scanning electron microscopy (SEM) (Phenom XL Desktop SEM, Thermo Fisher Scientific Inc., Waltham, MA, USA). Temperature-dependent dielectric properties, including dielectric constant (*ε*_r_) and dielectric loss (tan *δ*), were measured using a precision impedance analyzer (WK6500P, Wayne Kerr Electronic Instrument Co., London, UK) coupled with a temperature- control system (DMS-2000, Partulab Technology Co., Wuhan, China) over a range of −100 °C to 170 °C. Ferroelectric (*P*–*E* loops) and strain properties (*S*–*E* curves) were simultaneously acquired at 1 Hz using a ferroelectric tester (TF Analyzer 2000, aix-ACCT Inc., Aachen, Germany) equipped with an integrated laser displacement sensor. The strain data was accurately captured via an integrated laser displacement sensor. All measurements were performed on unpoled, as-sintered samples with an optimized thickness of 0.7–0.8 mm.

## 3. Results and Discussions

### 3.1. Phase Structure and Microstructure

**Figure 1:**
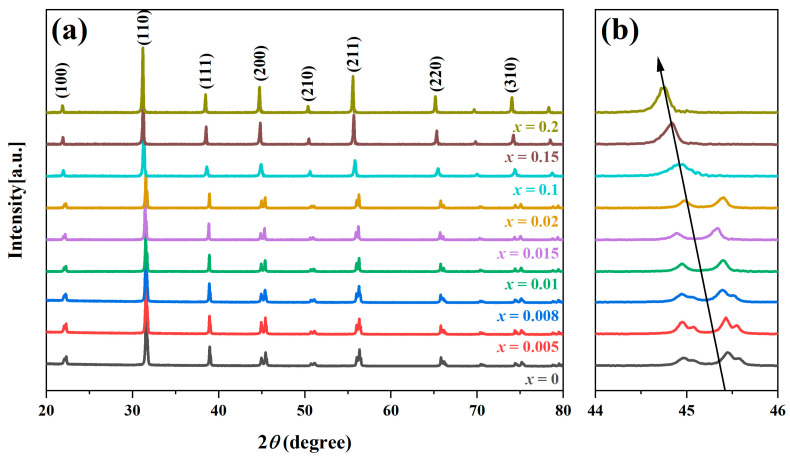
XRD patterns of BT-*x*BZ-BZN ceramics: (**a**) 20° ≤ 2*θ* ≤ 80°; (**b**) 44° ≤ 2*θ* ≤ 46°. The arrows indicate the shift of the diffraction peaks with increasing x.

Room-temperature XRD patterns of BT-*x*BZ-BZN ceramics are illustrated in Figure 1. All diffraction peaks can be indexed to a pure perovskite structure, and no secondary phases were detected within the scanned range. This confirms that BaZrO_3_ (BZ) and Bi(Zn_2/3_Nb_1/3_)O_3_ (BZN) have successfully diffused into the BaTiO_3_ lattice, forming a homogeneous solid solution. As shown in the magnified view in Figure 1b, the diffraction peaks shift toward lower 2*θ* angles with the introduction of BZ. This phenomenon is attributed to unit cell expansion caused by the substitution of larger Zr^4+^ ions (*r* = 72 pm) for smaller Ti^4+^ ions (*r* = 60.5 pm). For the undoped sample (*x* = 0), the distinct splitting of the (200) peak confirms a ferroelectric tetragonal (T) phase at room temperature, which is consistent with previous reports [40,41]. As *x* increases, the tetragonal distortion (c/a ratio) is progressively weakened due to the isotropic nature of the Zr^4+^ ion, leading to the merging of split peaks (*x* ≥ 0.1). The evolution of the (200) peak profile suggests a structural transition from T to pseudocubic (PC) symmetry.

**Figure 2:**
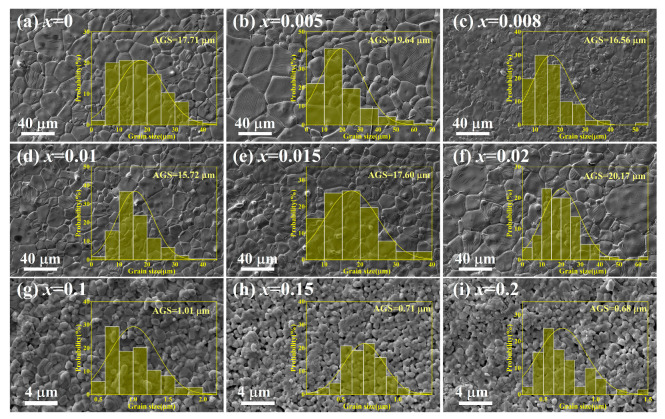
The surface scanning electron microscopy (SEM) image of BT-*x*BZ-BZN ceramics.

Figure 2 illustrates the surface morphologies and grain size distributions of the BT-*x*BZ-BZN ceramics. The undoped sample (*x* = 0) exhibits a well-developed coarse-grained structure with an average grain size (AGS) of 15.71 μm and clearly defined grain boundaries. While low -concentration Zr^4+^ substitution (*x* ≤ 0.02) maintains the AGS within a comparable range of 15–20 μm, a dramatic microstructural transition is observed upon further doping. At *x* ≥ 0.1, the AGS undergoes a sharp reduction by over an order of magnitude, plummeting to 1.01 μm for *x* = 0.1 and further to 0.66 μm for *x* = 0.2. This significant inhibition of grain growth is primarily attributed to the “solute drag” effect, wherein the high-concentration substitution of complex B-site ions increases the energetic barrier for grain boundary migration during the sintering process. For these heavily doped compositions, the grain boundaries become increasingly blurred, evolving into a fused and highly integrated texture. Such morphological evolution suggests that high dopant concentrations may induce a localized liquid phase that facilitates grain coalescence and effectively eliminates intergranular gaps. It is evident that the B-site doping strategy effectively achieves a highly refined and dense microstructure, providing a consistent morphological basis for subsequent physical investigations.

### 3.2. Dielectric Properties

**Figure 3:**
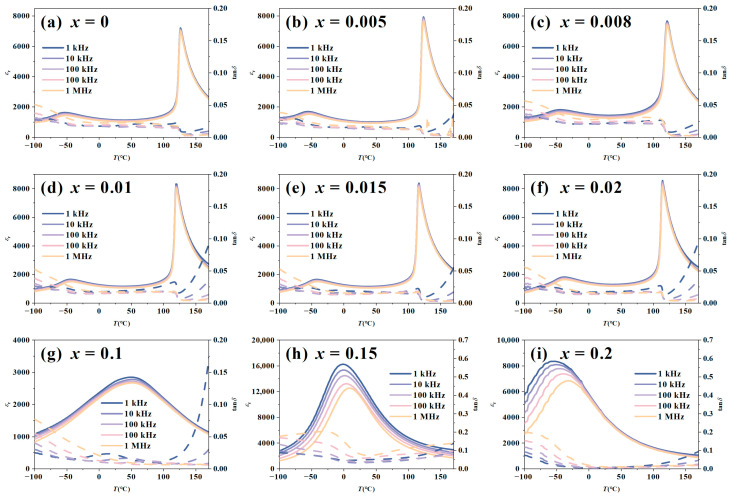
Temperature dependence of *ε*_r_ and tan*δ* for BT-*x*BZ-BZN ceramics.

Figure 3 presents the temperature dependence of the dielectric constant (*ε*_r_) and dielectric loss (tan*δ*) for the BT-*x*BZ-BZN ceramics measured at frequencies from 1 kHz to 1 MHz within the range of −100 °C to 170 °C. For low doping levels (0 ≤ *x* ≤ 0.02), two distinct dielectric anomalies are observed in the curves (Figure 3a–f). These correspond to the orthorhombic-tetragonal phase transition temperature (*T*_O–T_) and the tetragonal-cubic phase transition temperature (*T*_C_, Curie temperature), appearing in order of increasing temperature. For *x* = 0, *T*_O–T_ (−63 °C) is well below room temperature while *T*_C_ (126 °C) is above it, confirming the pure T phase at RT identified in XRD. With increasing *x* from 0.005 to 0.02, *T*_C_ decreases (123 °C → 110 °C) while *T*_O–T_ shifts upward (−52 °C → −38 °C). This convergence suggests a destabilization of the T phase; the substitution of chemically more stable and larger Zr^4+^ ions reduces the tetragonality (c/a ratio) and disrupts the Ti-O-Ti chains necessary for coherent polarization. This weakens the long-range ferroelectric order, a phenomenon consistent with the “lattice distortion” and “compositional disordering” effects typically observed in chemically modified perovskites [42,43]. For high doping levels (*x* ≥ 0.1), the sharp phase transition peaks merge into a single, broadened dielectric maximum (*ε*_m_). As *x* increases from 0.1 to 0.15, *T*_m_ (temperature corresponding to *ε*_m_) approaches and eventually drops below room temperature (~50 °C → −7 °C). This behavior indicates that high concentrations of Zr^4+^ disrupt the long-range ferroelectric order, promoting the formation of polar nanoregions (PNRs). This leads to characteristic relaxor ferroelectric behavior, manifesting as a diffuse phase transition and a macroscopically pseudocubic structure.

**Figure 4:**
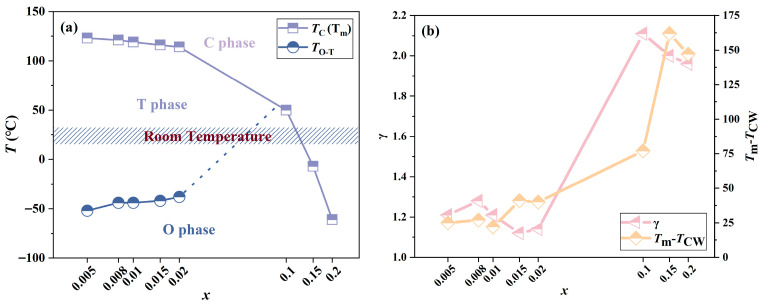
(**a**) Phase diagram and (**b**) composition dependence of *γ* and *T*_m_-*T*_CW_ for BT-*x*BZ-BZN ceramics.

Figure 4a illustrates the phase diagram of the BT-*x*BZ-BZN ceramics, derived from the combined results of XRD and dielectric analysis (at 100 kHz). As the BZ content increases, the *T*_O–T_ shifts toward room temperature, while the *T*_C_ (or *T*_m_) simultaneously decreases. This convergence of phase boundaries governs the evolution of the room-temperature crystal structure. Consequently, the phase stability evolves with composition *x* as follows: a stable T phase is maintained for 0 ≤ *x* ≤ 0.02; a coexistence of T and PC phases emerges in the transitional range of 0.02 < *x* < 0.1; and finally, a PC (or C) phase is formed for *x* ≥ 0.1, where relaxor ferroelectric characteristics dominate due to the suppression of long-range order.

To further investigate the diffuse phase transition characteristics of the BT-*x*BZ-BZN ceramics, the Curie-Weiss law is employed and expressed as:

1/*ε*_r_ = (*T* − *T*_0_)/C (1)

where C represents the Curie-Weiss constant and *T*_0_ is the Curie-Weiss temperature. Figure S1 (Supplementary Materials) illustrates the temperature dependence of 1/*ε*_r_ for the BT-*x*BZ-BZN ceramics. It is observed that the low-doped samples exhibit sharp dielectric peaks, characteristic of a typical long-range ferroelectric transition. However, as the doping level increases (*x* ≥ 0.1), the curves display a marked deviation from the Curie-Weiss law starting at a temperature *T*_CW_ (often referred to as the Burns temperature *T*_B_), which is significantly higher than the temperature of the dielectric maximum (*T*_m_). The magnitude of this deviation, quantified as Δ*T*_m_ = *T*_CW_ − *T*_m_, increases monotonically with *x*, as shown in Figure 4b. This expansion of the deviation region indicates that the phase transition evolves from a sharp, long-range ordered transition into a broad, diffuse phase transition characteristic of relaxor ferroelectrics.

Furthermore, the modified Curie-Weiss law is employed to evaluate the diffuse phase transition behaviors of the BT-*x*BZ-BZN ceramics by fitting the dielectric response above *T*_m_, according to the following empirical relation:

1/*ε*_r_ − 1/*ε*_m_ = (*T* − *T*_m_)*^γ^*/*C* (2)

where *γ* characterizes the diffuseness degree (1 ≤ *γ* ≤ 2). The plots of ln(1/*ε*_r_ − 1/*ε*_m_) versus ln(*T* − *T*_m_) in Figure S1 demonstrate excellent linearity for all compositions. As the BZ content increases, *γ* initially incerases before exhibiting a slight decline (Figure 4b). Notably, in the range of 0.1 ≤ *x* ≤ 0.2, *γ* approaches the theoretical limit of 2, confirming the strong relaxor nature of the heavily doped ceramics. This evolution in dielectric behavior corroborates the structural transitions observed via XRD. The complex multi-site substitution—Bi^3+^ at the A-site (Ba^2+^) and by Zr^4+^, Zn^2+^, and Nb^5+^ at the B-site—(Ti^4+^) introduces pronounced compositional disorder and charge inhomogeneity. As evidenced by the XRD peak shifts, the incorporation of larger Zr^4+^ ions expands the lattice and generates local elastic fields. These factors act as random fields that disrupt the long-range ferroelectric ordering, fragmenting macroscopic domains into PNRs. The dynamic thermal evolution of these PNRs—nucleating at *T_CW_* and freezing around *T*_m_—results in the observed frequency dispersion and diffuse dielectric response [47]. Furthermore, the microstructural refinement observed at higher doping levels (Figure 2) likely intensifies the internal stress arising from lattice distortion. This effect further suppresses the long-range ferroelectric coupling, thereby stabilizing the relaxor state.

### 3.3. Ferroelectricity and Strain Properties

**Figure 5:**
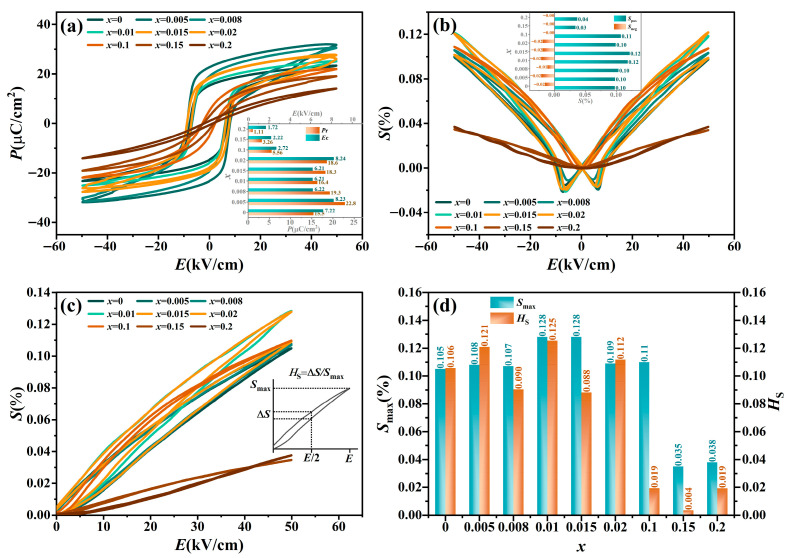
(**a**) Bipolar *P*–*E* loops (inset: *P*_r_ and *E*_C_), (**b**) bipolar *S*–*E* curves (inset: *S*_pos_ and *S*_neg_), (**c**) unipolar *S*–*E* curves (inset: *H*_S_), and (**d**) *S*_max_, *H*_S_ for BT-*x*BZ-BZN ceramics with different *x* contents. The *E*_max_ is 50 kV/cm and measuring frequency is 1 Hz.

Figure 5a,b display the composition-dependent bipolar polarization-electric field (*P–E*) and bipolar strain-electric field (*S*–*E*) loops for the BT-*x*BZ-BZN ceramics, measured at 1 Hz, 50 kV/cm, and room temperature. For compositions with 0 ≤ *x* ≤ 0.02, typical rectangular *P*–*E* loops and butterfly-shaped *S-E* curves are observed, signifying the dominance of long-range ferroelectric order. In contrast, as the BZ content increases to 0.1 ≤ *x* ≤ 0.2, both the *P*–*E* loops and *S*–*E* curves undergo a drastic transformation, becoming increasingly slender. Concurrently, both the remnant polarization (*P*_r_) and coercive field (*E*_c_) exhibit a marked decrease. This evolution is consistent with the structural and lattice dynamic analyses discussed earlier. The substitution of Zr^4+^ disrupts the long-range ferroelectric correlation, fragmenting macroscopic domains into non-interacting PNRs. These PNRs can easily reorient under an electric field but rapidly relax back to a random state upon field removal, naturally resulting in reduced hysteresis (low *E*_c_ and *P*_r_) [11,12]. Furthermore, the decrease in *E*_c_ aligns with the observed microstructural refinement (Figure 2); smaller grains induce higher internal stress constraints that suppress the formation of large, stable domains, facilitating the transition to a relaxor state.

Notably, for *x* ≥ 0.1, the negative strain (*S*_neg_) in the *S*–*E* curves vanishes, and the overall response becomes quasi-linear and nearly hysteresis-free. This indicates a fundamental shift in the strain mechanism from ferroelectric domain switching to electrostriction, characteristic of the PNR-dominated relaxor phase [18,19,20]. Especially, the ceramics with *x* = 0.1 present an optimal positive strain (*S*_pos_ ~ 0.11%) without *S*_neg_. In this relaxor phase, dynamic PNRs largely replace macroscopic domains. The reversible electric field-induced deformation and alignment of these PNRs minimize irreversible domain wall motion, thereby eliminating *S*_neg_ and resulting in a desirable quasi-linear, low-hysteresis strain response.

The unipolar *S*–*E* curves (Figure 5c) illustrate a trend where the strain initially increases and then decreases with increasing *x*. Figure 5d clearly shows the variation in maximum unipolar strain (*S*_max_) and strain hysteresis (*H*_S_) as a function of *x*. The *H*_S_ is defined as *H*_S_ = (∆*S*/*S*_max_) *×* 100%, where ∆*S* is the strain difference at half the maximum field [18,19,20]. The highest strain (0.128%) was obtained at *x* = 0.01 and 0.015, although this was accompanied by relatively high hysteresis. Significantly, an ultra-low hysteresis of 1.9% with a substantial strain of 0.11% was achieved at *x* = 0.1.

**Figure 6:**
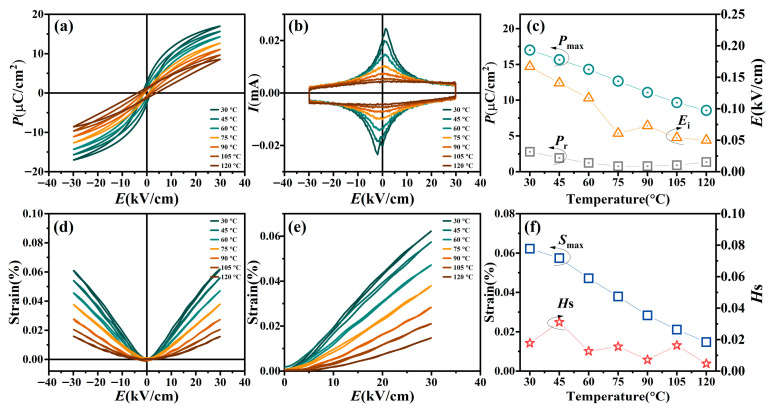
Temperature-dependent (**a**) bipolar *P*–*E* loops, (**b**) bipolar *I*–*E* curves, (**c**) *P*_r_, *P*_m_, and *E*_C_, (**d**) bipolar *S*–*E* curves, (**e**) unipolar *S*–*E* curves, and (**f**) *S*_max_ and *H*_S_ for BT-*x*BZ-BZN ceramics with *x* = 0.1. The *E*_max_ is 30 kV/cm and measuring frequency is 1 Hz.

Figure 6 illustrates the temperature evolution of ferroelectric, switching current, and strain properties for the BT-*x*BZ-BZN ceramics with *x* = 0.1, measured from 30 °C to 120 °C at a maximum electric field (*E*_max_) of 30 kV/cm and a frequency of 1 Hz. As shown in Figure 6a, the *P–E* loops exhibit a progressive slimming and tilting towards the electric field axis as temperature increases. This trend signifies a thermal degradation of the macroscopic ferroelectricity, which closely correlates with the dielectric phase transition temperature (*T*_m_ ~ 50 °C) identified in the dielectric spectra. The corresponding switching current (*I*–*E*) curves are plotted in Figure 6b. At 30 °C, the current loops display two distinct switching peaks that are notably asymmetric and offset from the zero-field axis, providing a direct experimental signature of internal bias field (*E*_i_). Additionally, the current at the maximum field remains at a low magnitude without a conspicuous exponential “upturn”, indicating that leakage current is negligible, and the samples maintain high insulation even at 120 °C. As plotted in Figure 6c, the maximum polarization (*P*_m_), *P*_r_, and *E*_i_ all decrease continuously with temperature. This reduction in polarization is primarily driven by thermal depolarization. At temperatures approaching and exceeding *T*_m_, the intensifying thermal fluctuations (*kT*) overcome the long-range Coulombic coupling forces among the PNRs [12,19,26]. Consequently, any residual ordered domain clusters disintegrate into dynamic, uncorrelated PNRs, leading to a rapid loss of macroscopic polarization.

The behavior of *E*_i_, defined as $E_{i}=||E_{C+}|-|E_{C-}||/2$, warrants closer examination. The origin of *E*_i_ is most likely attributed to the formation of defect-dipoles complexes (e.g., $\left(V_{O}^{\cdot \cdot }-A^{\prime \prime }{)}^{\times }\right.$ complexes), arising from the charge-compensating vacancies required by the aliovalent substitution of Bi^3+^ at the A-site and Nb^5+^/Zn^2+^ at the B-site [21,22,23]. At lower temperatures (*T* < *T*_m_), these defect dipoles align with the spontaneous polarization (*P*_s_) of the host domains, creating a microscopic built-in field that stabilizes the domain configuration and manifests as a shifted switching peak in the *I*-*E* curves. However, as the temperature rises (*T* > *T*_m_), the host lattice transforms into a paraelectric state and *P*_s_ vanishes, causing the defect dipoles to lose their alignment template. Combined with the enhanced thermal mobility of oxygen vacancies, this leads to a randomization of the defect dipoles, resulting in the thermal decay of *E*_i_ shown in Figure 6c.

Figure 6b depicts the corresponding bipolar and unipolar *S*–*E* curves. For the *x* = 0.1 composition, the room temperature bipolar strain curves exhibit a nearly hysteresis-free, parabolic-like shape. This indicates that ferroelectric domain switching contributes minimally to the overall strain, suggesting that the response is dominated by the electric field-induced alignment of PNRs rather than the irreversible motion of macroscopic ferroelectric domains [5,6,8]. With increasing temperature, the *S*–*E* curves become progressively more linear. As shown in Figure 6d, *S*_max_ decreases slowly below ~50 °C (close to *T*_m_) but drops precipitously above this temperature. This abrupt reduction is attributed to the crystallographic transformation towards a paraelectric state, where long-range *P*s vanishes. In this high-temperature regime, the lattice softens, and the cooperative interactions among PNRs are progressively lost due to enhanced thermal fluctuations. This behavior corresponds well with the rapid decrease in the dielectric constant peak observed in the dielectric spectra. Moreover, the *H*_S_ remains below 3% over the temperature range of 30–120 °C.

The transition from a butterfly-shaped loop to a strictly linear response at elevated temperatures confirms that the primary strain mechanism shifts away from ferroelectric domain reorientation. Instead, it becomes predominantly electrostrictive, wherein the strain is generated by the lattice deformation induced by the reversible polarization of PNRs. In the PNR-dominated relaxor phase, as temperature rises above *T*_m_, the size distribution of PNRs broadens, and the energy barrier for their reorientation decreases. This facilitates an increased proportion of electrically induced reversible switching, manifesting as the observed linearization of the *S*-*E* curves and the maintenance of a low remnant polarization. The absence of significant hysteresis and the increasingly linear response are hallmarks of efficient electrostrictive behavior, making these materials attractive for actuator applications requiring high linearity and low energy losses.

### 3.4. Electrostrictive Effect

**Figure 7:**
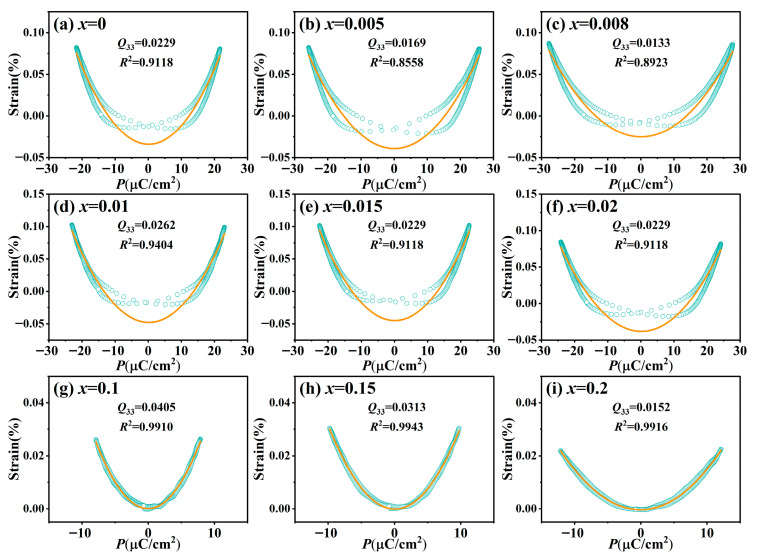
Bipolar *S*–*P* curves measured for BT-*x*BZ-BZN ceramics at room temperature. The *E*_max_ is 50 kV/cm and measuring frequency is 1 Hz. Open circles are the experimental data, while the solid bold lines are the fitting curves.

To quantitatively evaluate the electrostrictive effect in the BT-*x*BZ-BZN ceramics, the equation *S*_33_ = *Q*_33_*P*_3_^2^ is utilized, where *S*_33_, *Q*_33_, and *P*_3_ denote the longitudinal strain, longitudinal electrostrictive coefficient and polarization, respectively. Figure 7 illustrates the bipolar *S*–*P* curves derived from the corresponding bipolar *P–E* loops and bipolar *S*–*E* curves measured at room temperature. The electrostrictive coefficient *Q*_33_ was extracted from the quadratic coefficient of a polynomial fit to the *S*–*P* plots. A fitting accuracy (coefficient of determination, *R*^2^) is deemed satisfactory only when it reaches 0.95 or above [23]. For the undoped and low-doped ceramics (0 ≤ *x* ≤ 0.02), the *S*–*P* curve exhibits pronounced hysteresis, which is consistent with the behavior observed in their *P*–*E* and *S*–*E* curves. This hysteresis is primarily attributed to irreversible domain wall motion within the ferroelectric state, which significantly diminishes the fitting precision. Notably, as the BZ content increases into the relaxor region (0.1 ≤ *x* ≤ 0.2), the *S*–*P* curves demonstrate minimal hysteresis and achieve a high fitting accuracy (*R*^2^ > 0.99). At these compositions, the doping-induced pseudocubic phase weakens the long-range ferroelectric order (*P*_r_ ~ 1–5 μC/cm^2^), leading to a strain response that is predominantly electrostrictive. Simultaneously, the dynamic response of PNRs in the pseudocubic matrix becomes dominant, thereby reducing the energy loss associated with irreversible domain switching. For the *x* = 0.1 ceramic, *Q*_33_ reaches a pesk value of 0.0405 m^4^/C^2^, confirming its excellent electrostrictive properties. However, a further increase in *x* leads to a decline in *Q*_33_, which is likely attributed to the excessive dilution of PNRs and the increased concentration of defects introduced by high doping levels.

**Figure 8:**
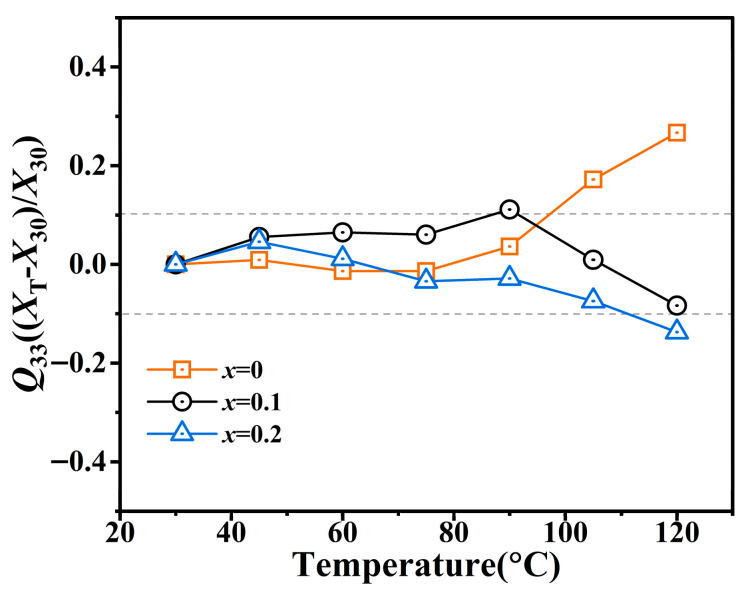
The temperature dependence of *Q*_33_ for BT-*x*BZ-BZN ceramics in the range of 30–120 °C. The *E*_max_ is 30 kV/cm and measuring frequency is 1 Hz.

Figure 8 depicts the temperature dependence of *Q*_33_ for the BT-*x*BZ-BZN ceramics (with *x* = 0, 0.1 and 0.2) from 30 °C to 120 °C. To ensure a rigorous and comparative stability analysis, all *Q*_33_ values were extracted from bipolar *P*–*E* loops and bipolar *S*–*E* curves (as Figure 6 for *x* = 0.1 and Figure S2 for other compositions) measured under a consistent *E*_max_ of 30 kV/cm and a frequency of 1 Hz. A notable observation is that for *x* = 0, *Q*_33_ remains relatively stable at lower temperatures but increases as the temperature approaches the Curie temperature (*T*_C_ ~ 110 –120 °C). This phenomenon is particularly intriguing: while the long-range ferroelectric order progressively disintegrates near *T*_C_ (or *T*_m_), short-range polarization fluctuations are enhanced by thermal disturbances, thereby contributing an additional electrostrictive effect. Furthermore, during the ferroelectric-paraelectric transition at *T*_C_, the softening of the elastic modulus leads to a more significant lattice deformation under the same electric field, thus elevating *Q*_33_.

In contrast, for *x* ≥ 0.1, *Q*_33_ exhibits a general decreasing trend with increasing temperature. In these pseudocubic compositions, isotropic thermal expansion randomizes the directions of lattice strain, consequently reducing the directional contribution of the electric field-induced strain. Specifically, for the *x* = 0.1 relaxor, where *T*_m_ is reduced to approximately 50 °C and the diffuse phase transition occurs over a broad temperature range, *Q*_33_ shows only a slight decrease while maintaining overall stability. The remarkable *Q*_33_ values and excellent thermal stability (with a variation of less than 10%) in the heavily doped compositions (*x* ≥ 0.1) are primarily attributed to the formation of a pseudocubic relaxor state and the dynamic response of PNRs. The slight reduction in *Q*_33_ at elevated temperatures likely stems from a combination of the thermal depolarization of PNRs, increased leakage current, and lattice expansion.

To further highlight the performance advantages, a comparison of the electromechanical properties of BT-0.1BZ-BZN with other BT-based lead-free ceramics is presented in Table 1. Notably, this work achieves an ultra-low hysteresis (*H*s = 1.9%) while maintaining a competitive unipolar strain (0.11% at 50 kV/cm), which is significantly superior to most reported ternary systems where *H*s typically exceeds 5%. Furthermore, while many existing materials exhibit *Q*_33_ values that are either lower in magnitude or highly temperature- sensitive, our system maintains a consistently high *Q*_33_ range (0.0371– 0.045 m^4^/C^2^) over a broad window (30–120 °C). This rare combination of high strain linearity and thermal robustness validates the effectiveness of the B-site doping strategy in engineering high-performance electrostrictive materials.

## 4. Conclusions

A systematic investigation of ferroelectric and strain properties in Zr^4+^-substituted BaTiO_3_-based BT-*x*BZ-BZN ceramics (0 ≤ *x* ≤ 0.2) reveals a composition-driven evolution from a long-range tetragonal ferroelectric to a pseudocubic relaxor state dominated by polar nanoregions (PNRs). Structural and dielectric analyses confirm that the *x* = 0.10 composition exhibits the critical feature. Therefore, it demonstrates an optimal combination of high unipolar strain (*S*_max_ = 0.11% at 50 kV/cm), ultra-low hysteresis (*H*_S_ = 1.9%) and a high electrostrictive coefficient (*Q*_33_ = 0.0405 m^4^/C^2^) at room temperature. Notably, exceptional temperature stability was achieved, with *Q*_33_ varying by less than 10% between 30 °C and 120 °C. This robust performance is attributed to the dominant electrostrictive effect, which arises from the reversible, electric-field-induced deformation and alignment of dynamic PNRs, thereby minimizing irreversible domain switching and conferring superior thermal stability. These characteristics collectively position BT-*x*BZ-BZN ceramics with *x* = 0.1 as a highly promising lead-free candidate for high-precision, low-loss actuator applications, offering a compelling pathway for next-generation ferroelectric devices.

## Figures and Tables

**Table 1:** Comparison of *S*_max,_ *H*s, and *Q*_33_ values among BT-based lead-free ceramics.

System	*E*_max_ (kV/cm)	*S*_max_ (%) (@RT)	*H*s (%) (@RT)	*Q*_33_ (m^4^/C^2^)	Measuring Temperature	Ref.
BT-0.1BZ-BZN	50	0.11	1.9	0.0371–0.045	30–120 °C	This Work
BT-0.08Bi(Li_0_._5_Nb_0.5_)O_3_	50	0.024	<10	0.037–0.049	30–120 °C	[14]
(Ba_0.9_Sr_0.1_)TiO_3_	60	0.2	<8	0.0409–0.479	30–120 °C	[15]
BCZT-0.06Bi	100	0.105	— ^1^	0.0223–0.0265	30–120 °C	[16]
Ba_0.94_(Li_0.5_Ho_0.5_)_0.06_TiO_3_	60	0.12	5	0.05–0.06	23–150 °C	[17]
Ba(Ti_0.98_Sn_0.02_)O_3_	40	0.08	<8	0.0398–0.0515	30–100 °C	[18]
0.5%La^3+^-doped Ba(Zr_0.2_Ti_0.8_)O_3_	60	0.08	<10	0.0427–0.0537	30–120 °C	[22]

^1^ “—” indicates the values were not provided in the cited references.

## Data Availability

The original contributions presented in this study are included in the article/Supplementary Material. Further inquiries can be directed to the corresponding author.

## Supplementary Materials

The following supporting information can be downloaded at: https://www.mdpi.com/article/10.3390/ma19020374/s1, Figure S1: Plots of 1/*ε*_r_ versus *T* for BT-*x*BZ-BZN ceramics; the inset displays the ln(1/*ε*_r_ − 1/*ε*_m_) versus ln(*T* − *T*_m_)) curves.; Figure S2: Temperature-dependent ferroelectric, strain and electrostrictive properties for BT-*x*BZ-BZN ceramics with *x* = 0 and 0.2.
