# Supplementary material for: Composition-Driven Ultra-Low Hysteresis Electrostrictive Strain in BaTiO3-BaZrO3-Bi(Zn2/3Nb1/3)O3 Ceramics with High Thermal Stability
Source: Materials (Basel). 2026 Jan 16;19(2):374. doi: 10.3390/ma19020374 (PMC12842765; doi:10.3390/ma19020374)
Supplement: Supplementary file 1 [file materials-19-00374-s001.zip › materials-4080309-supplementary.pdf]

Supplementary Materials

Composition-driven ultra-low hysteresis electrostrictive strain in $BaTiO_3$-$BaZrO_3$-$Bi(Zn_{2/3}Nb_{1/3})O_3$ ceramics with high thermal stability

Xuyi Yang [1], Qinyi Chen [1], Qilong Xiao [3], Qiang Yang [3], Wenjuan Wu [1,3], Bo Wu [2], Hong Tao [2], Junjie Li [1,3,*], Xing Zhang [1,3] and Yi Guo [1,3]

[1] Information Materials and Device Applications Key Laboratory of Sichuan Provincial Universities, Chengdu University of Information Technology, Chengdu 610225, China
[2] Sichuan Province Key Laboratory of Information Materials, Southwest Minzu University, Chengdu, 610041, Sichuan, PR China
[3] Optoelectronic Sensor Devices and Systems Key Laboratory of Sichuan Provincial Universities, College of Optoelectronic Engineering (Chengdu IC Valley Industrial College), Chengdu University of Information Technology, Chengdu 610225, China
* Correspondence: lijunjie@cuit.edu.cn

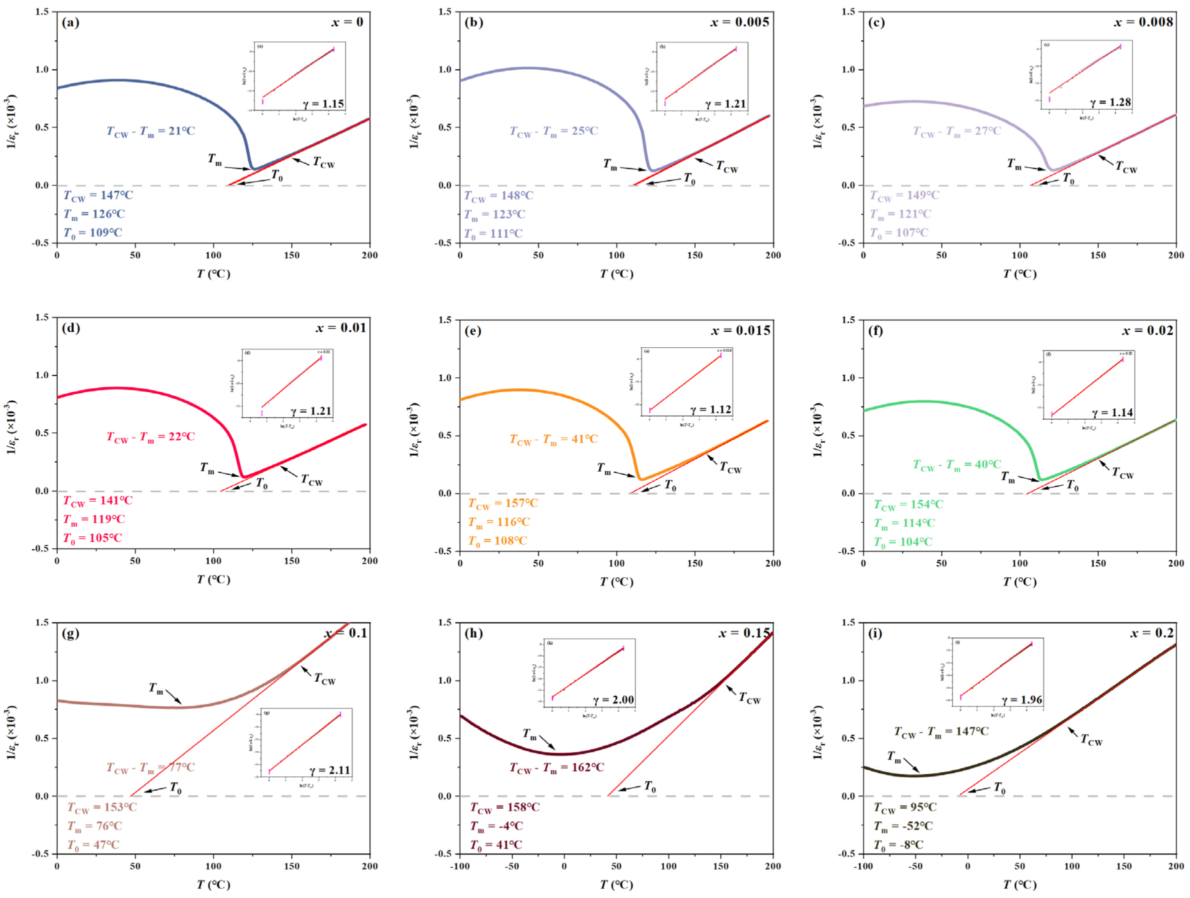

Figure S1. Plots of $1/\varepsilon_r$ versus T for BT-xBZ-BZN ceramics; the inset displays the $\ln(1/\varepsilon_r - 1/\varepsilon_m)$ versus $\ln(T - T_m)$ curves.

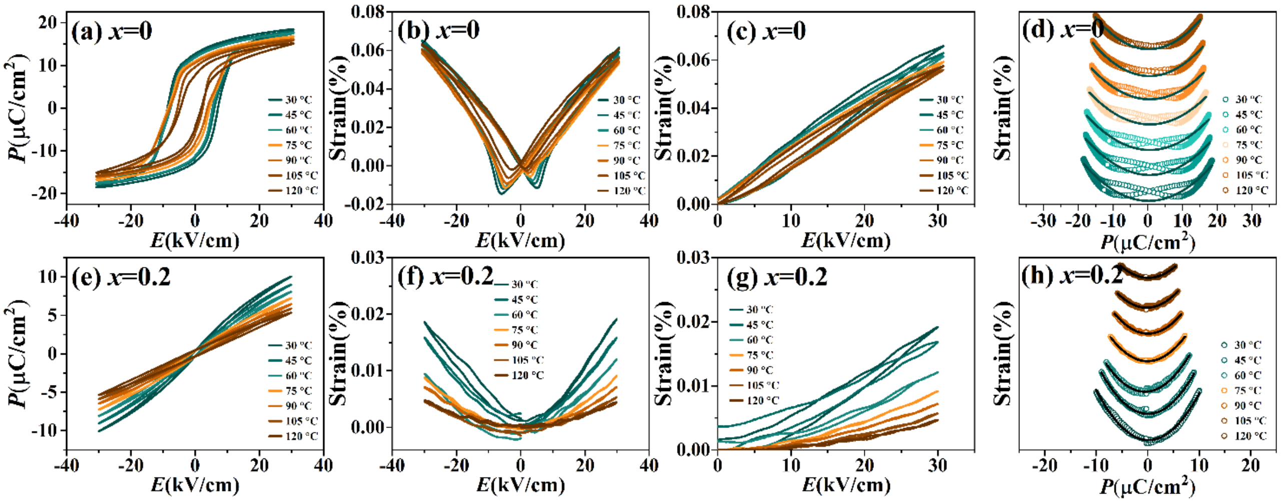

Figure S2. Temperature-dependent ferroelectric, strain and electrostrictive properties for BT-*x*BZ-BZN ceramics with *x*=0 and 0.2: (a, e) bipolar *P*–*E* loops; (b, f) bipolar *S*–*E* curves; (c, g) unipolar *S*–*E* curves; (d, h) *S*–*P* curves measured at 30 kV/cm and 1 Hz.
